# MYC upregulation as candidate predictive biomarker for radioresistance in cervical cancer

**DOI:** 10.1007/s11604-026-02036-z

**Published:** 2026-07-17

**Authors:** Kento Tomizawa, Soichiro Sakamoto, Yuhei Miyasaka, Takahiro Oike, Hidenari Hirata, Yuya Yoshimoto, Yasushi Sasaki, Takashi Tokino, Ken Ando, Atsushi Shibata, Tatsuya Ohno

**Affiliations:** 1 Department of Radiation Oncology, Gunma University Graduate School of Medicine, Maebashi, Japan; 2 Gunma University Heavy Ion Medical Center, 3-39-22, Showa-machi, Maebashi, Gunma 371-8511 Japan; 3 Department of Radiation Oncology, National Cancer Center Hospital East, Kashiwa, Japan; 4 Department of Radiation Oncology, Fukushima Medical University, Fukushima, Japan; 5 Center for Medical Education, Sapporo Medical University, Sapporo, Japan; 6 Research Institute for Frontier Medicine, Sapporo Medical University, Sapporo, Japan; 7 Division of Molecular Oncological Pharmacy, Graduate School of Pharmaceutical Sciences, Keio University, Tokyo, Japan

**Keywords:** Squamous cell carcinoma of the cervix, Radiotherapy, Radioresistance, MYC, RNA sequencing, Copy number alteration

## Abstract

**Background:**

A subset of uterine cervical cancers recurs locally after definitive radiotherapy, highlighting the need for establishing biomarkers to predict radioresistance for dose optimisation. However, low local recurrence rate constitutes a barrier to research progress. This study aimed at exploring candidate predictive biomarker of radioresistance of uterine cervical cancer by analyzing multiple omics layers using nested case-control approach.

**Methods:**

A discovery cohort (*n* = 8) was created from a series of 192 patients (all with squamous cell carcinoma of the uterine cervix underwent definitive radiotherapy) by 1:1 random case-control matching on pelvic recurrence (PR) for RNA sequencing. A validation cohort (*n* = 54) was created from the same series by 1:2 propensity score matching on PR for panel-based DNA sequencing and pelvic recurrence-free survival (PRFS) analysis. by using the Kaplan–Meier method and cause-specific proportional hazards models.

**Results:**

In the discovery cohort, Gene set enrichment analysis revealed that the genes targeted by *MYC* were upregulated in patients with PR. In the validation cohort, the prevalence of *MYC* amplification was significantly greater in patients with PR than in those without (72% vs. 36%, respectively). The 5-year PRFS of patients with *MYC* amplification was significantly worse than that of those without (47.4% vs. 80.6%, respectively). Multivariate analysis identified *MYC* amplification as the significant predictor of PR.

**Conclusions:**

These data suggest that upregulation of MYC plays a role in radioresistance of uterine cervical cancer, making *MYC* amplification a potential predictive biomarker for radioresistance.

**Supplementary Information:**

The online version contains supplementary material available at https://doi.org/10.1007/s11604-026-02036-z.

## Introduction

Cervical cancer occurs in approximately 0.6 million women worldwide annually, accounting for approximately 0.3 million deaths [1]. The standard treatment for locally advanced cervical cancer is combined chemoradiotherapy and brachytherapy [2]. Although treatment outcomes have improved dramatically owing to technological advancements in image-guided adaptive brachytherapy (IGABT) [3, 4], some patients experience local recurrence. For example, the International Study Including External Beam and Magnetic Resonance Imaging (MRI)-guided Brachytherapy in Cervical Cancer (EMBRACE-I) reported a local recurrence rate of approximately 10% within 5 years [3]. Studies in which patients were treated with external beam radiotherapy (EBRT) with a central shielding technique, and computed tomography (CT)-based IGABT, a treatment regimen predominant in Asia region, report comparable rates of local recurrence [4, 5]. Dose-volume analyses of patients treated with these regimens reveal that recurrence occurs from a subset of tumors irradiated with a sufficient dose, suggesting that such tumors harbor intrinsic radioresistance [6, 7]. Therefore, if we are to optimise the radiation dose to overcome this, we need to identify predictive biomarkers of radioresistance.

Several studies have explored radioresistance biomarkers for cervical cancer (Supplementary Table 1); however, most investigated a few genes of interest using a single analytical method. Thus, a more comprehensive approach that covers multiple omics layers may be more successful in identifying the mechanism(s) underlying radioresistance of cancer cells. Another obstacle to pursuing this research goal is small statistical power, mainly due to low local recurrence rates for the disease, which makes it difficult to perform costly bioanalysis on an entire cohort. Here, we aimed to identify biomarker predictive of radioresistance of cervical cancer. To address the above-mentioned issues, we used a nested case-control approach to analyze gene expression and copy number alterations in samples obtained from pretreated tumors.

## Materials and methods

### Study cohorts

**Fig. 1:**
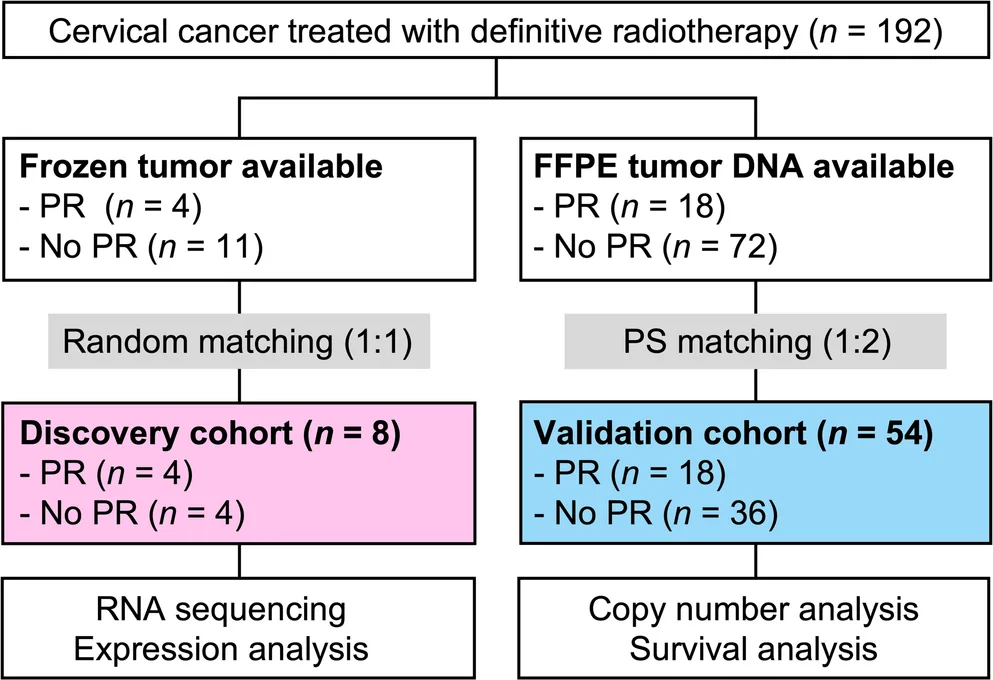
Flow diagram showing generation of the study cohorts and the analyses performed. FFPE = formalin-fixed and paraffin-embedded; PR = pelvic recurrence; PS = propensity score

One-hundred-and-ninety-two patients with newly diagnosed and pathologically confirmed squamous cell carcinoma of the uterine cervix, treated with definitive radiotherapy at the Gunma University Hospital (Maebashi, Gunma, Japan) during 2006–2013, were analysed retrospectively. Among them, a discovery cohort of patients for whom pretreatment frozen tumor samples were available (*n* = 15) was created by performing 1:1 random matching between patients who developed pelvic recurrence (PR), defined as post-treatment tumor regrowth or recurrence in the pelvic region [8] (*n* = 4), and those who did not (*n* = 11) (Fig. 1). In addition, a validation cohort of patients for whom DNA samples extracted from pretreatment formalin-fixed and paraffin-embedded (FFPE) tumor biopsy specimens that passed quality checks for sequencing [8] were available was created using nested case-control approach; in this case, 1:2 propensity score matching between patients with PR (*n* = 18) and those without (*n* = 72) was performed (Fig. 1). During propensity score matching, tumor volume (as assessed by pretreatment MRI), nodal status, and the number of chemotherapy cycles were used as covariates. Random matching and propensity score matching were performed using the modules within R (version 4.3.2, R Foundation for Statistical Computing, Vienna, Austria), namely *sample* and *matchit* (method: *nearest*), respectively. The study was approved by the Institutional Ethical Review Committee of Gunma University Hospital (approval number, 1109). All patients provided written informed consent.

### Treatment

Definitive radiotherapy, comprising three-dimensional conformal EBRT and in-room CT-based high-dose-rate IGABT, was performed as described in a previous study [4]. Briefly, EBRT comprised 50 Gy, delivered to the whole pelvis in 25 fractions, where the last 20 Gy was delivered using the central shielding technique (3 cm in width). Patients with nodal involvement received boost irradiation to the nodes (6–10 Gy in 3–5 fractions). IGABT was performed using Fletcher-Suit Asian Pacific applicators. Trocar point needles were used for asymmetric tumors to optimise dose distribution. Using an ^192^Ir remote-after-loading system, 24 Gy was delivered to the D_90_ high-risk clinical target volume or to the point A in four sessions (one session per week) in all cases. Concurrent weekly cisplatin (40 mg/m^2^) was administered to patients with stage III–IV disease, a maximum tumor diameter ³ 4 cm, or nodal involvement.

### Follow-up

Patients were followed up every 1–3 months for the first 2 years after treatment, every 3–6 months for the next 3 years, and then every 6–12 months thereafter. Disease status was assessed at each follow-up by gynecological examination and imaging (CT or MRI). PR-free survival (PRFS), calculated from the date of initiation of radiotherapy to the date of PR or death from any cause, was recorded [8, 9].

### RNA sequencing

Total RNA was extracted from frozen tumor samples using a RNeasy Kit (QIAGEN, Venlo, Holland). RNA was shipped to DNA Chip Research Inc. (Tokyo, Japan) and subjected to sequencing using the NextSeq500 platform (Illumina Inc., San Diego, CA, U.S.A.; approximately 20 million 75-bp paired-end reads). Transcripts were annotated using Ensembl (2014.01.02). Normalisation was performed using the trimmed mean of M-values method.

### Gene set enrichment analysis (GSEA)

GSEA was performed using GSEA 4.2.3 software [10]. GSEA is a method used to evaluate the upregulation or downregulation of specific biological functions by comparing genome-wide gene expression between two groups in terms of predefined sets of functionally related genes, namely gene sets. To this end, all genes are ranked according to the magnitude of differential expression between the two groups, and each gene set is then tested to determine whether it is enriched with top- or bottom-ranked genes. By this approach, GSEA can detect the upregulation or downregulation of biological functions even when the expression changes of individual genes are modest. In GSEA, the normalized enrichment score (NES) is used as an indicator of the degree of enrichment of top- or bottom-ranked genes within a given gene set. In addition, the false discovery rate (FDR) is applied to control for type I error in the context of multiple testing. Although there are no universally established cutoff values for NES and FDR in GSEA, we adopted thresholds of an NES of 1.5 and an FDR of 0.2, which have been widely used in previous studies. Regarding gene set, the hallmark genes set (v2024.1, MSigDB, Broad Institute-hosted human annotations) were used [11]. Gene expression in patients with PR was evaluated relative to patients without PR as the reference, using 1,000 permutations (all other parameters were set to default).

### Copy number variation (CNV) analysis

CNV was assessed as descried in a previous study [12]. Briefly, DNA was extracted from the FFPE tumor biopsy specimens using the QIAamp DNA FFPE Tissue kit (Qiagen) and subjected to multiplex PCR amplification using an Ion AmpliSeq Comprehensive Cancer Panel (Thermo Fisher Scientific, Waltham, MA, U.S.A.). Sequencing was performed using an Ion Torrent sequencer (Thermo) and the data were aligned to the Genome Reference Consortium Human Build 37 (hg19). Reads were counted using Torrent Suite (version 5.0, Thermo). CNVs were determined by Ion Reporter software (version 5.0, Thermo) using an algorithm based on a Hidden Markov Model. A copy number ≥ 3 was defined as amplification.

### Public omics data analysis

RNA sequencing and copy number data (namely Expression Public 24Q4 and Copy Number Public 24Q4, respectively) for cancer cells registered in Cancer Cell Line Encyclopedia (CCLE) were obtained from DepMap portal website (https://depmap.org/portal/, last accessed on January 7th, 2025). RNA sequencing, copy number, and patient survival data were retrieved from The Cancer Genome Atlas Program (TCGA)-Cervical Squamous Cell, Adenosquamous Carcinoma, and Adenocarcinoma (CESC; cBioPortal website at https://www.cbioportal.org/, last accessed on January 20th, 2025). To analyze the TCGA-CESC dataset, information about radiotherapy was obtained from the Broad Institute’s Firehose website (https://gdac.broadinstitute.org/, last accessed on January 20th, 2025).

### Statistical analysis

Mann–Whitney U test and Fisher’s exact test were used to compare numerical variables and categorical variables, respectively. Pearson’s correlation test was used to examine correlations between gene expression and copy number. Overall survival (OS) and PRFS were assessed using the Kaplan–Meier method, and data were analysed using the log-rank test. A cause-specific proportional hazards models was created to assess the utility of variables for predicting PRFS. The proportional hazards for each variable were assessed using the Schoenfeld residual test, performed using EZR [13]. All other analyses were performed using GraphPad Prism (version 10, GraphPad Software, San Diego, CA, USA). Differences were considered statistically significant at *p* < 0.05.

## Results

To identify biological factors associated with recurrence arising from definitive radiotherapy for uterine cervical cancer (i.e., clinical radioresistance), we generated two nested case-control cohorts, i.e., a discovery cohort (*n* = 8, by 1:1 matching) and a validation cohort (*n* = 54, by 1:2 matching), from patients with locally advanced uterine cervical cancer treated and followed up using standard protocols (Fig. 1). First, we performed RNA sequencing of pretreatment tumor biopsy specimens from the discovery cohort, and found no significant differences in age, the 2018 International Federation of Gynecology and Obstetrics (FIGO) stage, pretreatment tumor volume, pelvic or para-aortic lymph node status, or the presence of concurrent chemotherapy between patients with PR and those without (Table 1); all patients were followed up until they developed PR, or for more than 5 years.

**Table 1:** Patient characteristics for the discovery cohort

Characteristics		PR-positive				PR-negative				*p* value
	Pt:	1	2	3	4	5	6	7	8	
Age, years		58	56	54	43	61	74	45	33	0.88
FIGO stage		IIIC1	IIIC1	IIB	IB3	IIIC1	IIIB	IIIC1	IIIC1	0.43
Tumor volume (cc)		61	102	63	66	27	24	78	41	0.20
Pelvic LN involvement		+	+	−	−	+	−	+	+	> 0.99
PALN involvement		−	−	−	−	−	−	−	−	> 0.99
Concurrent chemotherapy		+	+	+	+	+	+	+	+	> 0.99

FIGO = the 2018 International Federation of Gynecology and Obstetrics; LN = lymph node; PALN = para-aortic lymph node; PR = pelvic recurrence.

*p* values for the differences between PR-positive and -negative patients, assessed by Mann–Whitney U test and Fisher’s exact test for comparison of numerical variables and categorical variables, respectively, are shown.

**Fig. 2:**
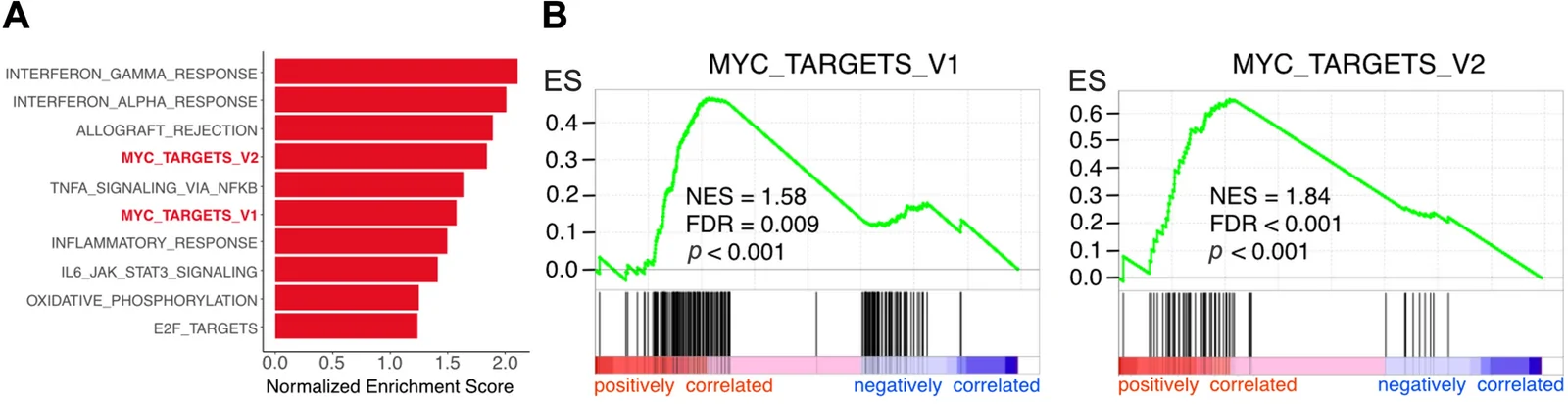
Upregulation of MYC signaling in patients in the discovery cohort that developed post-radiotherapy pelvic recurrence. (**A**) Gene Set Enrichment Analysis using the hallmark gene sets. The top 10 gene sets significantly upregulated in patients with pelvic recurrence (PR) compared those without are shown. Cutoff values of 1.5 for the normalized enrichment score (NES) and 0.2 for the false discovery rate (FDR) were applied. (**B**) Enrichment plots for the MYC targets-V1 and -V2 gene sets, demonstrating that genes included in these gene sets, i.e., target of *MYC* genes, are significantly enriched in PR cases

Figure 2A shows the top 10 gene sets significantly upregulated in patients with PR relative to those without as assessed by GSEA. The significantly upregulated gene sets were associated with inflammatory responses, allograft rejection, oxidative phosphorylation, and MYC signaling. Among them, we focused on MYC signaling because MYC is a transcription factor that regulates the expression of numerous genes including those involved in cancer cell proliferation, metabolism, and protein synthesis [14]; and the finding that both gene sets composed of MYC target genes (i.e., V1 and V2) were significantly upregulated in patients with PR suggests enhanced MYC activity itself (Fig. 2B).

**Fig. 3:**
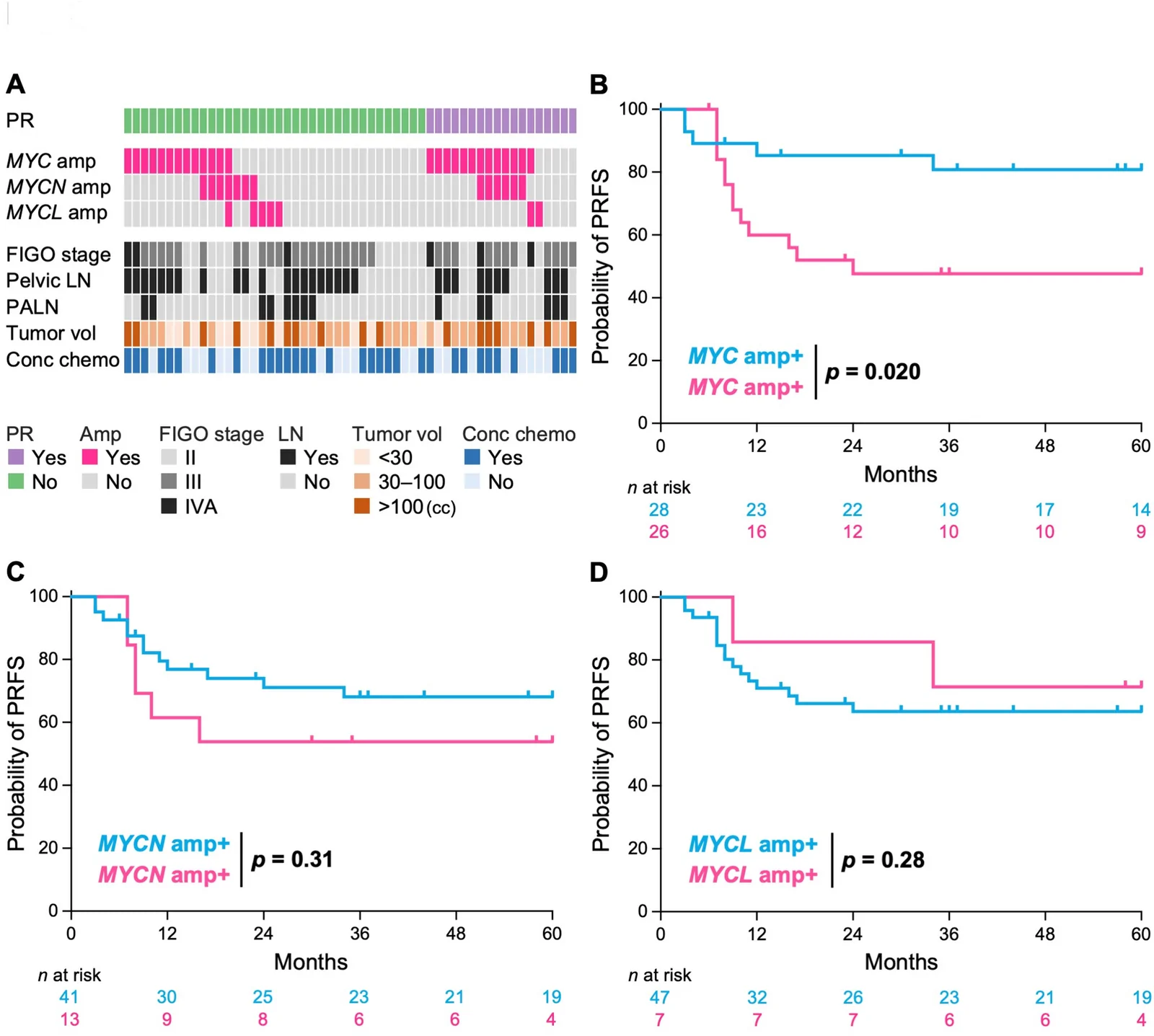
*MYC* amplification is associated with pelvic recurrence in the validation cohort. (**A**) Summary of the amplification status of *MYC*, *MYCN*, and *MYCL*, and clinical parameters. (**B**–**D**) Pelvic recurrence-free survival according to amplification of *MYC* (**B**), *MYCN* (**C**), or *MYCL* (**D**), calculated using the Kaplan–Meier method. Amp = amplification; Conc chemo = concurrent chemotherapy; FIGO = the 2018 International Federation of Gynecology and Obstetrics; LN = lymph node; PALN = para-aortic lymph node; PR = pelvic recurrence; PRFS = pelvic recurrence-free survival; vol = volume. *p* values assessed by the log-rank test are shown

Analysis of the CCLE and TCGA-CESC datasets revealed that high expression of *MYC* was associated significantly with *MYC* amplification (Supplementary Fig. 1). Thus, we next examined whether *MYC* amplification is associated with clinical radioresistance of uterine cervical cancer by analyzing the validation cohort. Along with *MYC*, we investigated amplification of *MYCN* and *MYCL* because these genes share target genes with *MYC* [14]. In this cohort, there was no significant difference in age, FIGO stage, pretreatment tumor volume, pelvic or para-aortic lymph node status, or the presence of concurrent chemotherapy, between patients with PR and those without (Table 2). Among the PR patients, 14 experienced recurrences in the primary tumor and 4 in the pelvic lymph nodes. The median follow-up period was 17 months (range, 4–73 months) for patients with PR, and 69 months (range, 7–131 months) for those without. Interestingly, *MYC* amplification was significantly more prevalent in patients with PR than in those without (72% [13/18 patients] vs. 36% [13/36 patients], respectively; *p* = 0.020; Table 2; Fig. 3A). Patients with *MYC* amplification had significantly worse 5-year PRFS than those without: 47.4% (95% confidence interval [CI]: 27.2%–65.1%) vs. 80.6% (95% CI: 59.3%–91.2%), respectively; Fig. 3B). The statistical significance of the results remained unchanged when the PR patients were limited to those who experienced recurrence from the primary tumor (Supplementary Fig. 2). Furthermore, univariate and multivariate analyses identified *MYC* amplification as the only significant predictor of PR (hazard ratio [HR] = 3.21 [95% CI: 1.21–10.06], *p* = 0.026; and HR = 3.10 [95% CI: 1.13–9.89], *p* = 0.036, respectively) among the parameters examined: i.e., *MYC* amplification, pretreatment tumor volume, pelvic lymph node status, and the presence of concurrent chemotherapy (Table 3, Supplementary Fig. 3). There was no significant difference in age, FIGO stage, pretreatment tumor volume, pelvic or para-aortic lymph node status, or the presence of concurrent chemotherapy, between MYC amplification-positive and -negative patients (Supplementary Table 2). By contrast, there was no significant difference in the prevalence of *MYCN* amplification and *MYCL* amplification between patients with PR and those without (33% [6/18 patients] vs. 19% [7/36 patients], *p* = 0.31; and 11% [2/18 patients] vs. 14% [5/36 patients], *p* > 0.99, respectively; Table 2). Consistent with this, the amplification status of *MYCN* or *MYCL* had no effect on PRFS (*p* = 0.31 and 0.28, respectively) (Figs. 3C, D). There was a modest trend toward worse PRFS for patients who were positive for amplification of either *MYC*, *MYCN*, or *MYCL* (*p* = 0.16) than those negative for these amplifications (Supplementary Fig. 4). In the TCGA-CESC cohort, high expression of *MYC*, but not that of *MYCN* or *MYCL*, was associated significantly with poor OS post-radiotherapy (Supplementary Fig. 5). Taken together, these data indicate that MYC upregulation is associated with clinical radioresistance of uterine cervical cancer.

**Table 2:** Patient characteristics for the validation cohort

Characteristics	Total patients	PR-positive	PR-negative	*p* value
	(*n* = 54)	(*n* = 18)	(*n* = 36)	
Age, years	63 (35–87)	58 (35–87)	66 (36–82)	0.39
FIGO stage				0.53
II	16 (30%)	4 (22%)	12 (33%)	
III	32 (59%)	11 (61%)	21 (59%)	
IVA	6 (11%)	3 (17%)	3 (8%)	
Tumor volume (cc)	63 (6–477)	64 (6–421)	60 (8–477)	0.40
Pelvic LN involvement				> 0.99
Positive	30 (56%)	10 (56%)	20 (56%)	
Negative	24 (44%)	8 (44%)	16 (44%)	
PALN involvement				0.51
Positive	14 (26%)	6 (33%)	8 (22%)	
Negative	40 (74%)	12 (67%)	28 (78%)	
Concurrent chemotherapy				0.77
Yes	32 (59%)	10 (56%)	22 (61%)	
No	22 (41%)	8 (44%)	14 (39%)	
*MYC* amplification				**0.020**
Yes	26 (48%)	13 (72%)	13 (36%)	
No	28 (52%)	5 (28%)	23 (64%)	
*MYCN* amplification				0.31
Yes	13 (24%)	6 (33%)	7 (19%)	
No	41 (76%)	12 (67%)	29 (81%)	
*MYCL* amplification				> 0.99
Yes	7 (13%)	2 (11%)	5 (14%)	
No	47 (87%)	16 (89%)	31 (86%)	

FIGO = the 2018 International Federation of Gynecology and Obstetrics; LN = lymph node; PALN = para-aortic lymph node; PR = pelvic recurrence.

For age and tumor volume, median (range) is shown. *p* values for the differences between PR-positive and -negative patients, assessed by Mann–Whitney U test and Fisher’s exact test for comparison of numerical variables and categorical variables, respectively, are shown

**Table 3:** Univariate and multivariate analyses for pelvic recurrence

Variables	Univariate		Multivariate	
	HR (95%CI)	*p* value	HR (95%CI)	*p* value
Tumor volume	1.00 (0.99–1.01)	0.39	1.00 (0.99–1.01)	0.53
Pelvic LN involvement	1.13 (0.44–2.98)	0.78	0.90 (0.32–2.50)	0.83
MYC amplification	3.21 (1.20–10.06)	**0.026**	3.17 (1.17–10.02)	**0.030**

CI = confidence interval; HR = hazard ratio; LN = lymph node. p values assessed by cause-specific proportional hazards models are shown.

## Discussion

This study aimed to elucidate the biological factors associated with recurrence in patients with cervical cancer treated with definitive photon radiotherapy, which generally demonstrates favorable treatment outcomes. In this treatment, the primary tumor and pelvic lymph nodes are subjected to highly heterogeneous dose fractionation and dose rates, resulting from a complex combination of external beam radiotherapy and brachytherapy in terms of both irradiation field and schedule. It is highly challenging to reproducibly model this complex treatment scheme in in vitro or in vivo experimental systems; in this sense, such clinically relevant radioresistance cannot, in theory, be elucidated by utilizing standard in vitro or in vivo systems. From this standpoint, we explored the omics features associated with this phenotype in the real-world settings. By analyzing multiple omics layers using a nested case-control approach, we demonstrated for the first time that MYC upregulation plays a role in clinical radioresistance of uterine cervical cancer. Amplification in *MYC*, but not that of its family members *MYCN* or *MYCL*, predicted a worse prognosis with respect to pelvic control after definitive radiotherapy. Because *MYC* copy number can be assessed using commercially available gene panel tests, our findings can be validated in real-world settings.

*MYC* is a well-studied driver oncogene that is dysregulated in more than 70% of human cancers [15]. *MYC* contributes to disease progression, metastatic potential, and resistance to anticancer agents, in many types of cancer [16]; however, there are few reports that examined involvement of *MYC* in resistance to radiotherapy. Hirata et al. performed whole exome sequencing of esophageal squamous cell carcinoma treated with definitive chemoradiotherapy, and reported that the *MYC* copy number increased in patients who did not respond to treatment [17]. Zafarana et al. performed copy number analysis of *MYC*, and reported that *MYC* gain was associated with biochemical relapse in patients with prostate cancer treated with radiotherapy [18]. Taken together with our own findings, the data suggest that MYC upregulation may be a pan-cancer biomarker for radioresistance.

The mechanism by which *MYC* contributes to radioresistance remains unclear. However, several potential pathways have been suggested. First, *MYC* promotes repair of DNA damage induced by irradiation. Studies show that *MYC* activates transcription of genes involved in double-strand break repair by binding to their promoter regions [19–21]. This mechanism itself is shared among normal and cancer cells; however, the increased MYC copy number observed in this study suggests enhanced MYC activity in these tumors. In general, cancer cells exhibit vulnerabilities in cell cycle checkpoints, which increases the risk of mitotic catastrophe [22]. From this perspective, MYC upregulation could compensate for this vulnerability by promoting the repair of radiation-induced DNA damage. Second, it is considered that *MYC* is involved in maintaining cancer stem cells that exhibit radioresistance [23, 24]. MYC also regulates the factors associated with cancer stem cell properties such as p21^WAF1/CIP1^, cyclin D_1_, ABCG2, and ABCB5 [25, 26]. Third, *MYC* dysregulates antitumor immune responses [27]. Studies show that radiotherapy induces expression of major histocompatibility complex (MHC) class I molecules on the surface of cancer cells, leading to activation of type I interferon [28, 29], and downregulates expression of CD47 [30]; i.e., the ‘do-not-eat-me’ signal, for dendric cells [31]. From this perspective, MYC suppresses expression of MCH class I expression [32, 33] and upregulates that of CD47 [34]. Moreover, *MYC* increases glycolysis (i.e., the Warburg effect) [35, 36], thereby contributing to an immunosuppressive tumor microenvironment [37–40].

This study has the following limitations. First, cost constraints meant that we used a limited number of samples for gene the expression study; however, few previous studies have analysed the gene expression profile of uterine cervical cancer associated with PR post-radiotherapy. Therefore, the results could be a valuable indicator for development of personalised treatments for uterine cervical cancer. In addition, we performed copy number validation to compensate for the small number of samples used for the gene expression study. Second, because this was a single-center, retrospective study, we cannot rule out the possibility of selection bias; however, neither our discovery nor validation cohorts appeared to be heterogeneous in terms of patient background, which might affect clinical outcomes (Tables 1 and 2).

In summary, we demonstrate here that MYC upregulation plays a role in clinical radioresistance of uterine cervical cancer. The predictive significance of *MYC* amplification in the context of PR warrants prospective validation if we are to develop precision medicine approaches to increase the efficacy of radiotherapy for uterine cervical cancer.

Supplementary Fig. 1. *MYC* expression according to *MYC* amplification status. (A) CCLE dataset. (B) TCGA-CESC dataset. TPM = transcripts per million. *p* values assessed by Mann–Whitney U test are shown.

Supplementary Fig. 2. Fisher exact test (A) and log-rank test (B) for pelvic recurrence-free survival (PRFS) stratified by *MYC* amplification status in validation cohort by limiting pelvic recurrence (PR) cases to those who experienced recurrence from the primary tumor (*n* = 14). Amp = amplification.

Supplementary Fig. 3. Proportional hazards for the variables used in the multivariate cause-specific proportional hazards models shown in Table 3, as assessed by Schoenfeld’s residuals test. (A) Tumor volume. (B) Pelvic lymph node involvement. (C) Concurrent chemotherapy. (D) *MYC* amplification. Amp = amplification; CT = chemotherapy; LN = lymph node.

Supplementary Fig. 4. Pelvic recurrence-free survival of patients who were positive for amplification of either *MYC*, *MYCN* or *MYCL* versus those who were not in the validation cohort, calculated using the Kaplan–Meier method. P values were calculated using the log-rank test. Amp = amplification; PRFS = pelvic recurrence-free survival.

Supplementary Fig. 5. Overall survival of the TCGA-CESC patients treated (left panels) or not (right panels) with radiotherapy, stratified according to *MYC* (upper panels), *MYCN* (middle panels), or *MYCL* (lower panels) expression status. P values were calculated using the log-rank test. OS = overall survival.

## Supplementary Information

Below is the link to the electronic supplementary material.

Supplementary Material 1

## Data Availability

Research data are not available at this time.
