# Supplementary material for: MYC upregulation as candidate predictive biomarker for radioresistance in cervical cancer
Source: Jpn J Radiol. 2026 Jul 17;44(10):1854–62. doi: 10.1007/s11604-026-02036-z (PMC13623779; doi:10.1007/s11604-026-02036-z)
Supplement: Supplementary file 1 [file 11604_2026_2036_MOESM1_ESM.pdf]

Supplementary Table 1 Summary of translational studies reporting radioresistant biomarkers in cervical cancer

Biomarkers	Radioresistant status	Analysis method	Research strategy	Year	Authors	Journal
NSUN6	High expression	RNA seq	Exploratory	2024	Yu et al.	Mol Cancer
KLK5	High expression	Microarray	Exploratory	2022	Zhou et al.	Gynecol Oncol
p73	Low expression	Microarray	Exploratory	2004	Liu et al.	Clin Cancer Res
miRs signature	Signature	Microarray	Exploratory	2016	Pedroza-Torres et al.	Gynecol Oncol
TBM	High	DNA seq	Exploratory	2021	Ota et al.	Jpn J Radiol
KRAS, SMAD4	Mutation	DNA seq	Exploratory	2021	Oike et al.	Int J Mol Sci
FGFR	Mutation	DNA seq	Exploratory	2020	Yoshimoto et al.	Gynecol Oncol
STK11	Mutation	DNA seq	Exploratory	2020	Hirose et al.	Gynecol Oncol
ALDH1	High expression	IHC	Targeted	2023	Fahmi et al.	Asian Pac J Cancer Prev
CD163	High expression	IHC	Targeted	2023	Sheng et al.	Cancers
$CD8^{+}$ TILs	Low expression	IHC	Targeted	2021	Mori et al.	Oncol Let
P16INK4A	Low expression	IHC	Targeted	2018	Fu et al.	Int J Mol Sci
IGF-1R β	High expression	IHC	Targeted	2017	Moreno-Acosta et al.	Radiat Oncol
SKP2	High expression	IHC	Targeted	2017	Fu et al.	Oncotarget
HK2	High expression	IHC	Targeted	2015	Huang et al.	Int J Clin Exp Pathol
Osteopontin, E-cadherin	High or low expression	IHC	Targeted	2015	Huang et al.	J Histochem Cytochem
SOX2, OCT4	High expression	IHC	Targeted	2014	Shen et al.	J Histochem Cytochem

EGFR	High expression	IHC	Targeted	2011	Pérez-Regadera et al.	Am J Clin Oncol
DNAPK, Ku70, Ku86	High expression	IHC	Targeted	2009	Beskow et al.	Br J Cancer
Phosphorylated AKT	High expression	IHC	Targeted	2006	Kim et al.	Br J Cancer
Ku80	High expression	IHC	Targeted	2003	Harima et al.	Am J Clin Oncol
Stanniocalcin 2	High expression	IHC, qPCR	Targeted	2014	Shen et al.	Int J Clin Exp Pathol
miR-21	High expression	qPCR	Targeted	2024	Putra et al.	Int J Mol Sci
miR-326	Low expression	qPCR	Targeted	2022	Zhang et al.	Genes Genomics
hsa_circ_0009035	High expression	qPCR	Targeted	2021	Zhao et al.	Mol Cell Biol
PIK3CA	Mutation	qPCR	Targeted	2018	Lachkar et al.	Medicine
MALAT1	High expression	qPCR	Targeted	2016	Lu et al.	Tumour Biol
HOTAIR	High expression	qPCR	Targeted	2015	Li et al.	Tumour Biol
miR-21	High expression	qPCR	Targeted	2015	Liu et al.	Biochem Biophys Res Commun
miR-200a/-200b/-429	Low expression	qPCR, RNA seq	Targeted	2022	Nilsen et al.	Mol Oncol
PAX1	Hypomethylation	MS-qPCR	Targeted	2023	Shi et al.	Death Discov
ras p21	Mutation	ELISA	Targeted	1999	Sreelekha et al.	J Exp Clin Cancer Res

Abbreviations: ELISA = enzyme-linked immunosorbent assay, IHC = immunohistochemistry, MS = methylation specific, qPCR = quantitative polymerase chain reaction, seq = sequencing, TILs = tumor infiltrating lymphocytes, TMB = tumor mutational burden

Supplementary Table 2 Patient characteristics for the validation cohort according to *MYC* amplification status

**Characteristics**	**Total patients**	***MYC* amplification**		***p* value**
		Positive	***Negative***	
	**(*n* = 54)**	**(*n* = 26)**	**(*n* = 28)**	
Age, years	63 (35–87)	58.5 (35–82)	67 (36–87)	0.29
FIGO stage				0.15
II	16 (30%)	8 (31%)	8 (29%)	
III	32 (59%)	13 (50%)	19 (68%)	
IVA	6 (11%)	5 (19%)	1 (3%)	
Tumor volume (cc)	63 (6–477)	66 (9–477)	63 (6–421)	0.70
Pelvic LN involvement				0.79
Positive	30 (56%)	15 (58%)	15 (54%)	
Negative	24 (44%)	11 (42%)	13 (46%)	
PALN involvement				0.36
Positive	14 (26%)	5 (19%)	9 (32%)	
Negative	40 (74%)	21 (81%)	19 (68%)	
Concurrent chemotherapy				0.58
Yes	32 (59%)	14 (54%)	18 (64%)	
No	22 (41%)	8 (46%)	14 (36%)	

Abbreviation: FIGO = the 2018 International Federation of Gynecology and Obstetrics; LN = lymph node; PALN = para-aortic lymph node. For age and tumor volume, median (range) is shown. *p* values for the differences between *MYC* amplification-positive and -negative patients, assessed by Mann–Whitney U test and Fisher's exact test for comparison of numerical variables and categorical variables, respectively, are shown.

Supplementary Fig. 1

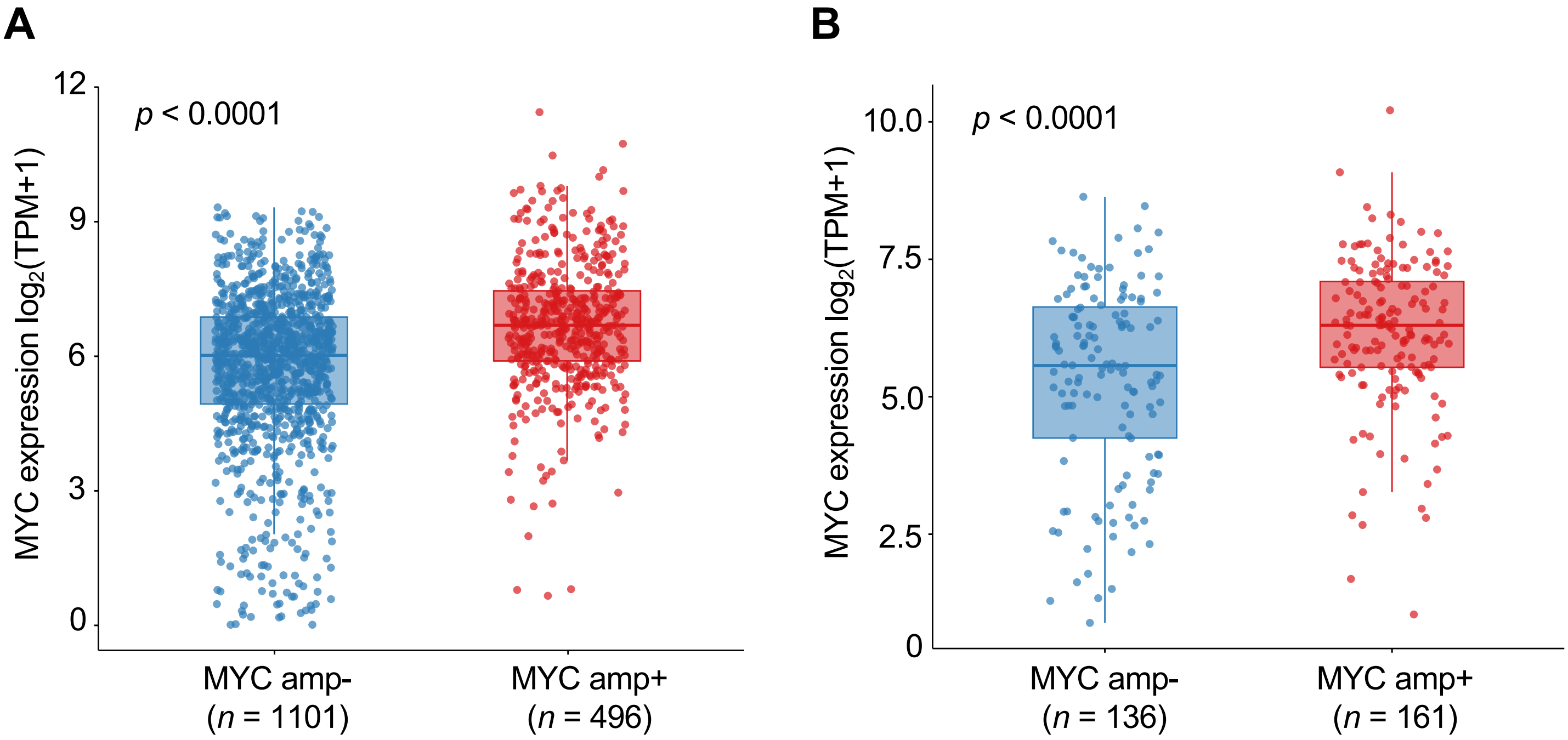

Supplementary Fig. 2

A

		MYC amp +	*MYC* amp −
PR	+	10	4
PR	−	13	23

***p* = 0.031**

B

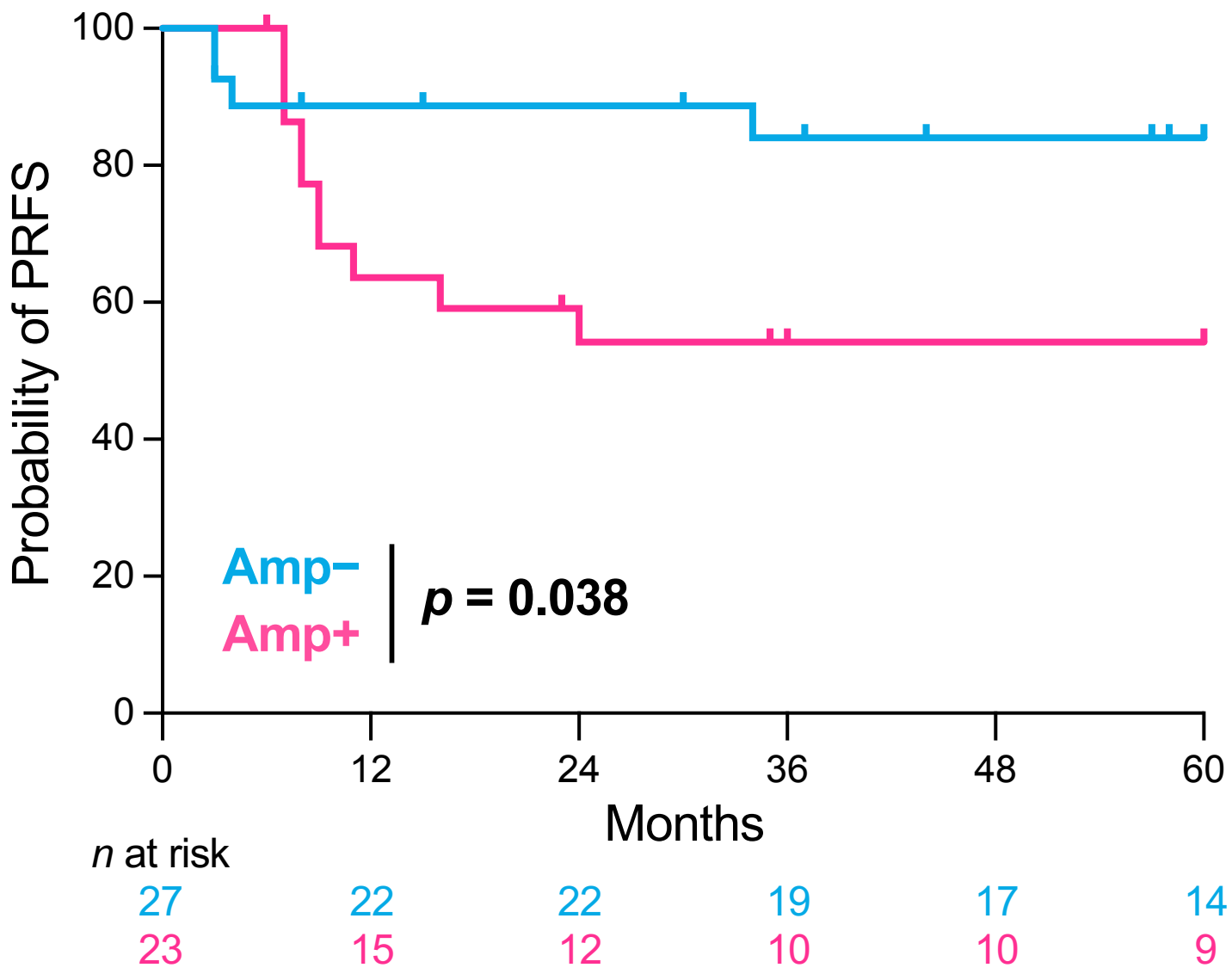

Supplementary Fig. 3

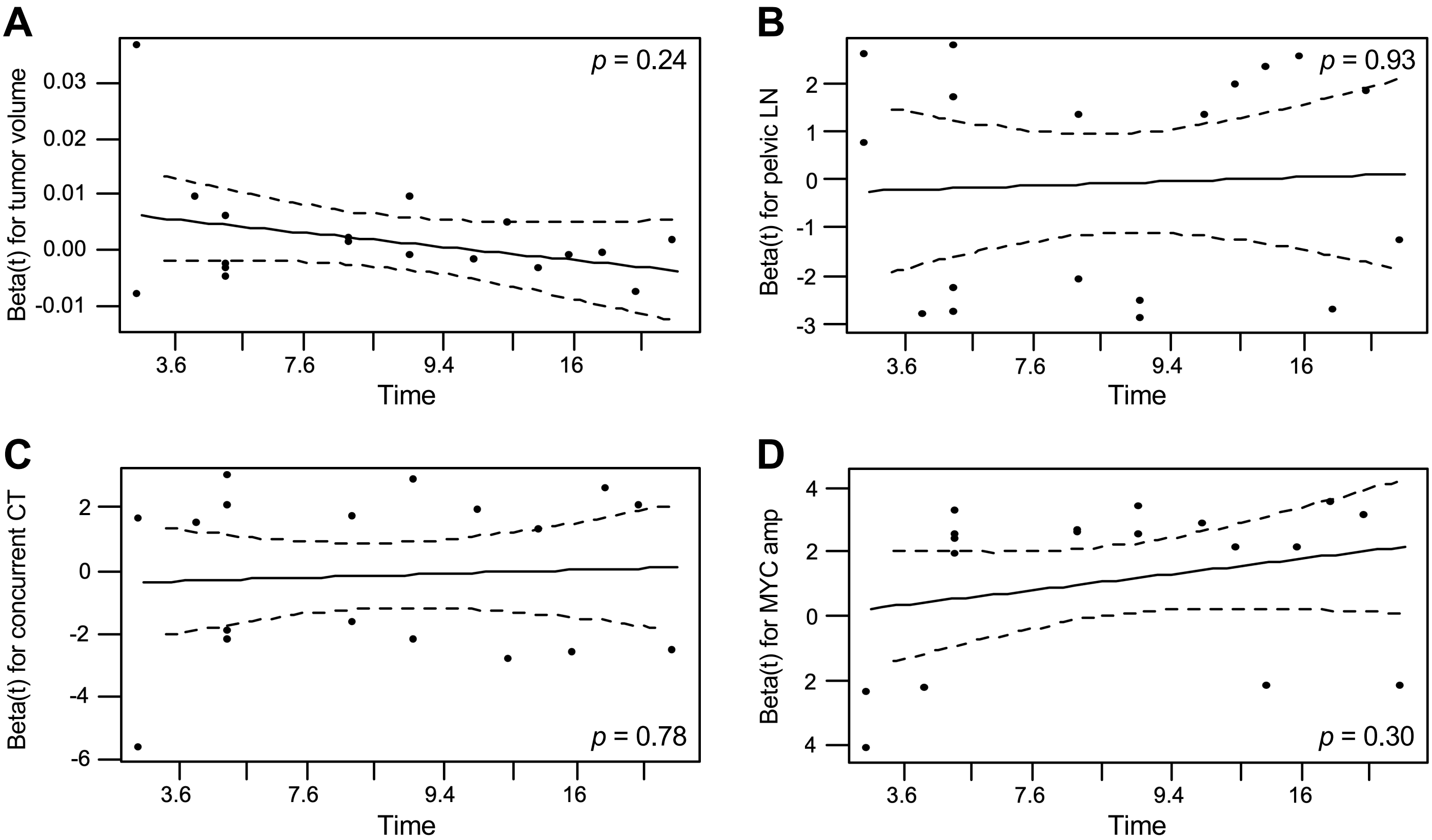

Supplementary Fig. 4

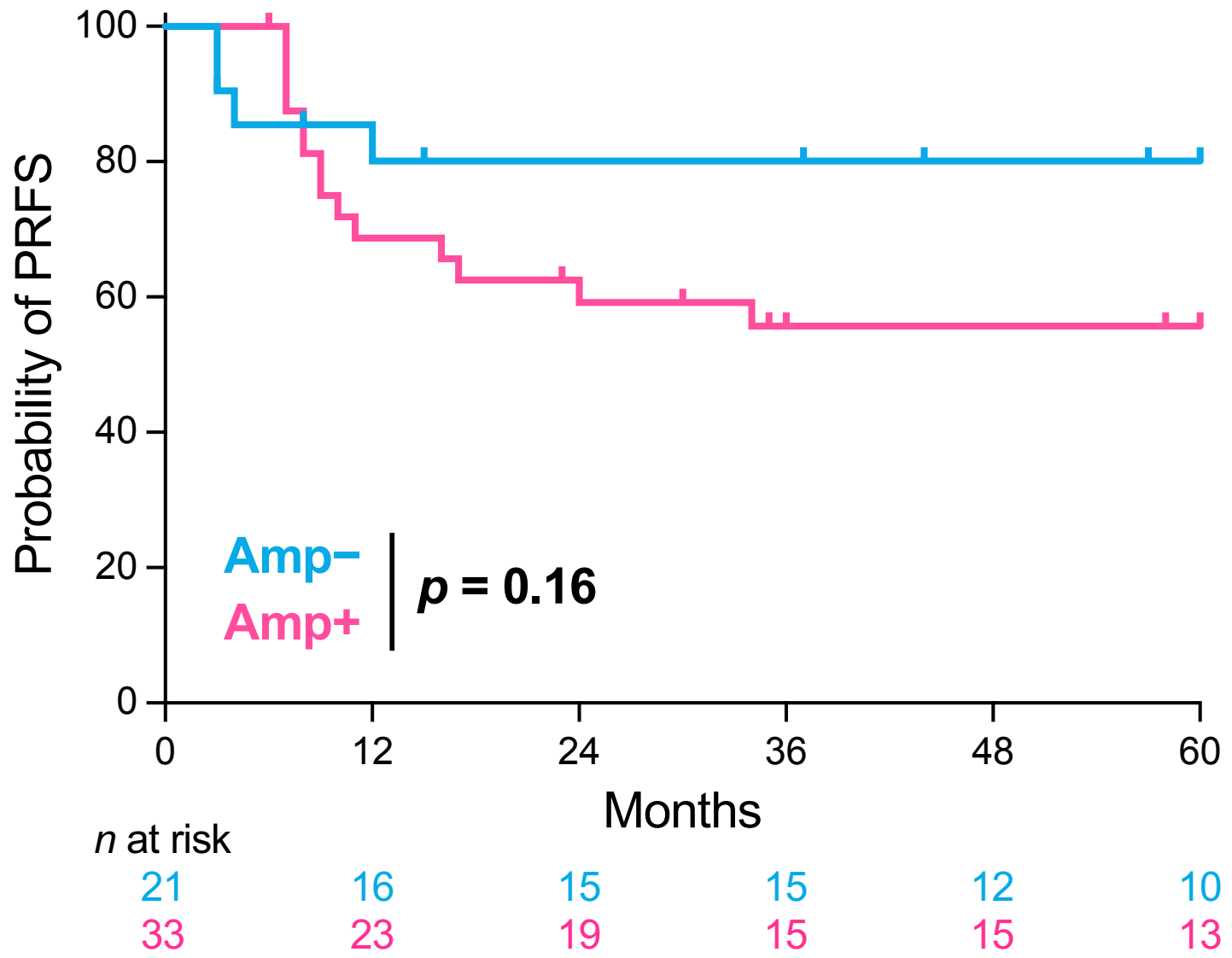

Supplementary Fig. 5

MYC

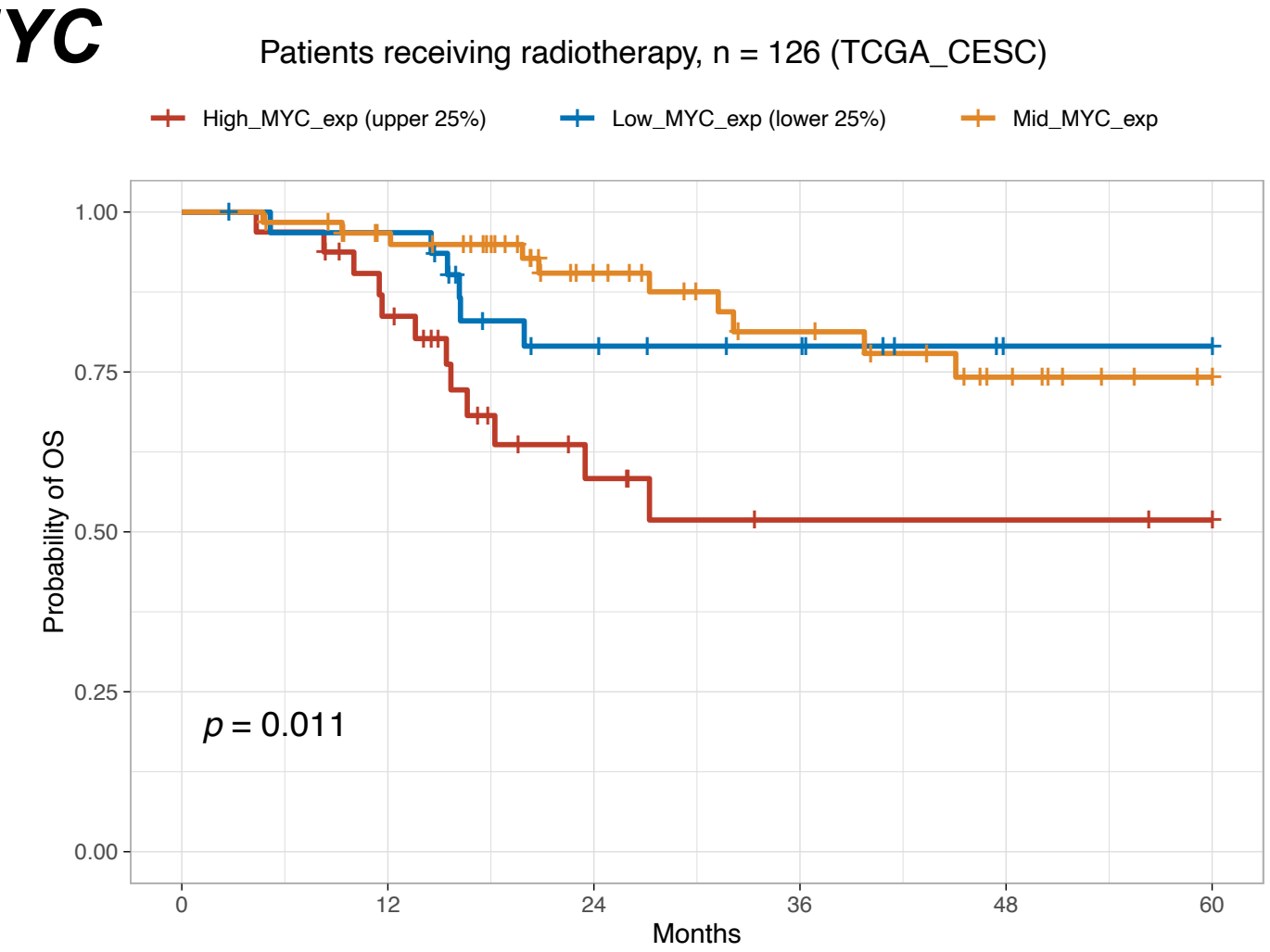

Number at risk

	0	12	24	36	48	60
High_MYC_exp(upper 25%)	32	25	11	7	7	6
Low_MYC_exp(lower 25%)	32	30	19	16	10	10
Mid_MYC_exp	62	54	34	25	17	10

Months

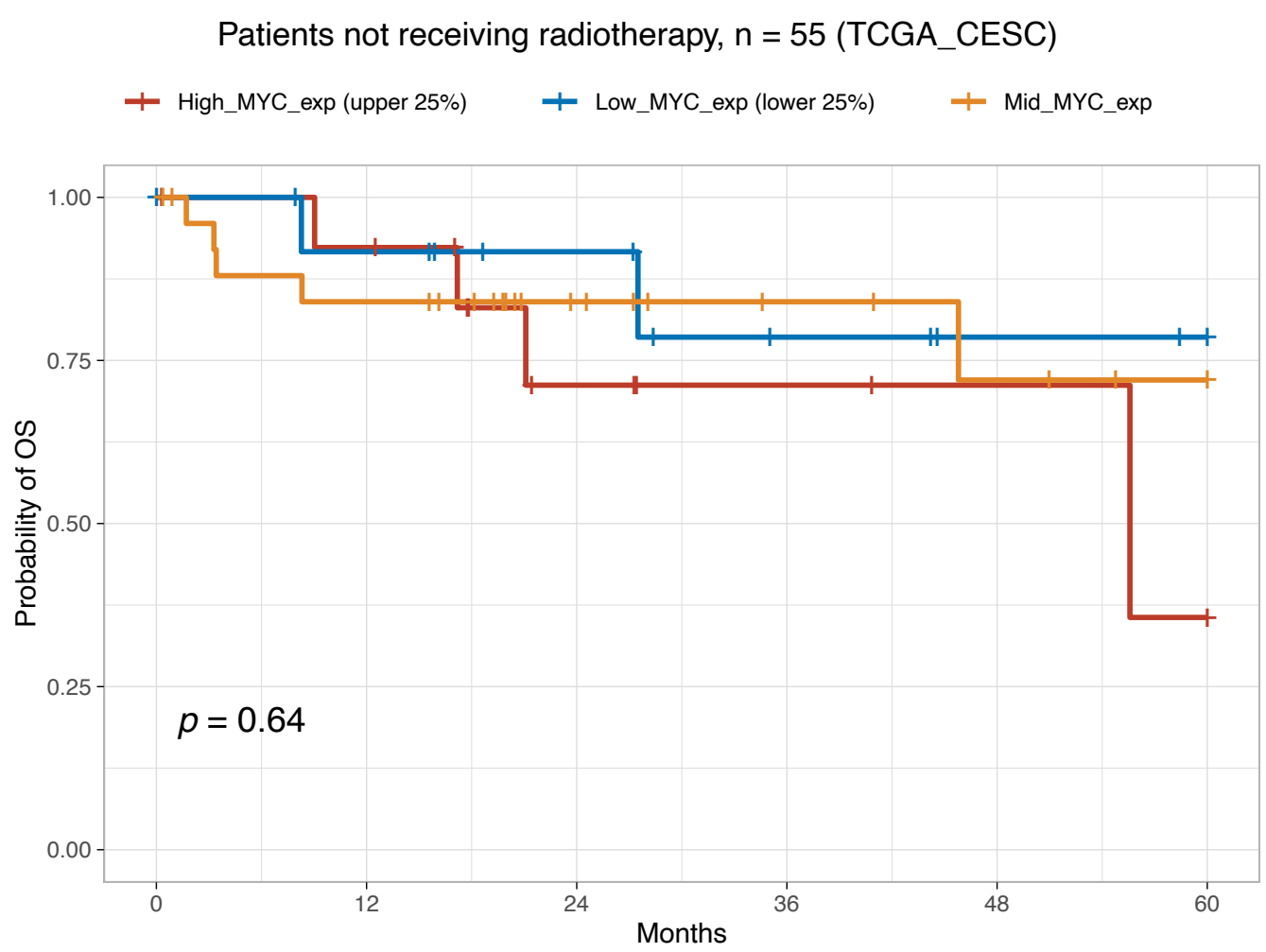

Number at risk

	0	12	24	36	48	60
High_MYC_exp(upper 25%)	14	12	5	3	2	1
Low_MYC_exp(lower 25%)	14	11	8	4	2	1
Mid_MYC_exp	27	21	12	8	6	4

Months

MYCN

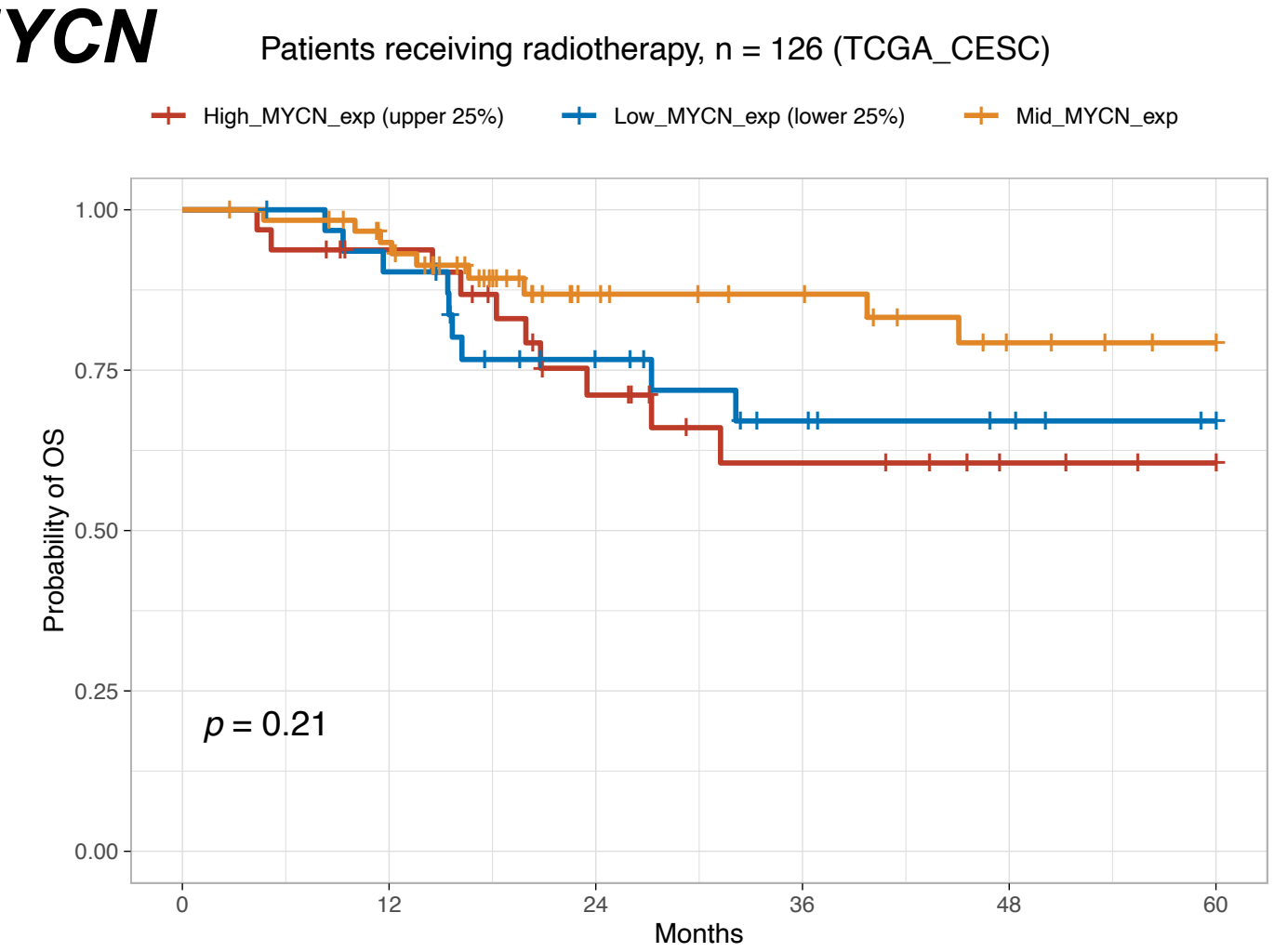

Number at risk

	0	12	24	36	48	60
High_MYCN_exp(upper 25%)	32	27	17	11	7	5
Low_MYCN_exp(lower 25%)	32	28	18	12	9	6
Mid_MYCN_exp	62	54	29	25	18	15

Months

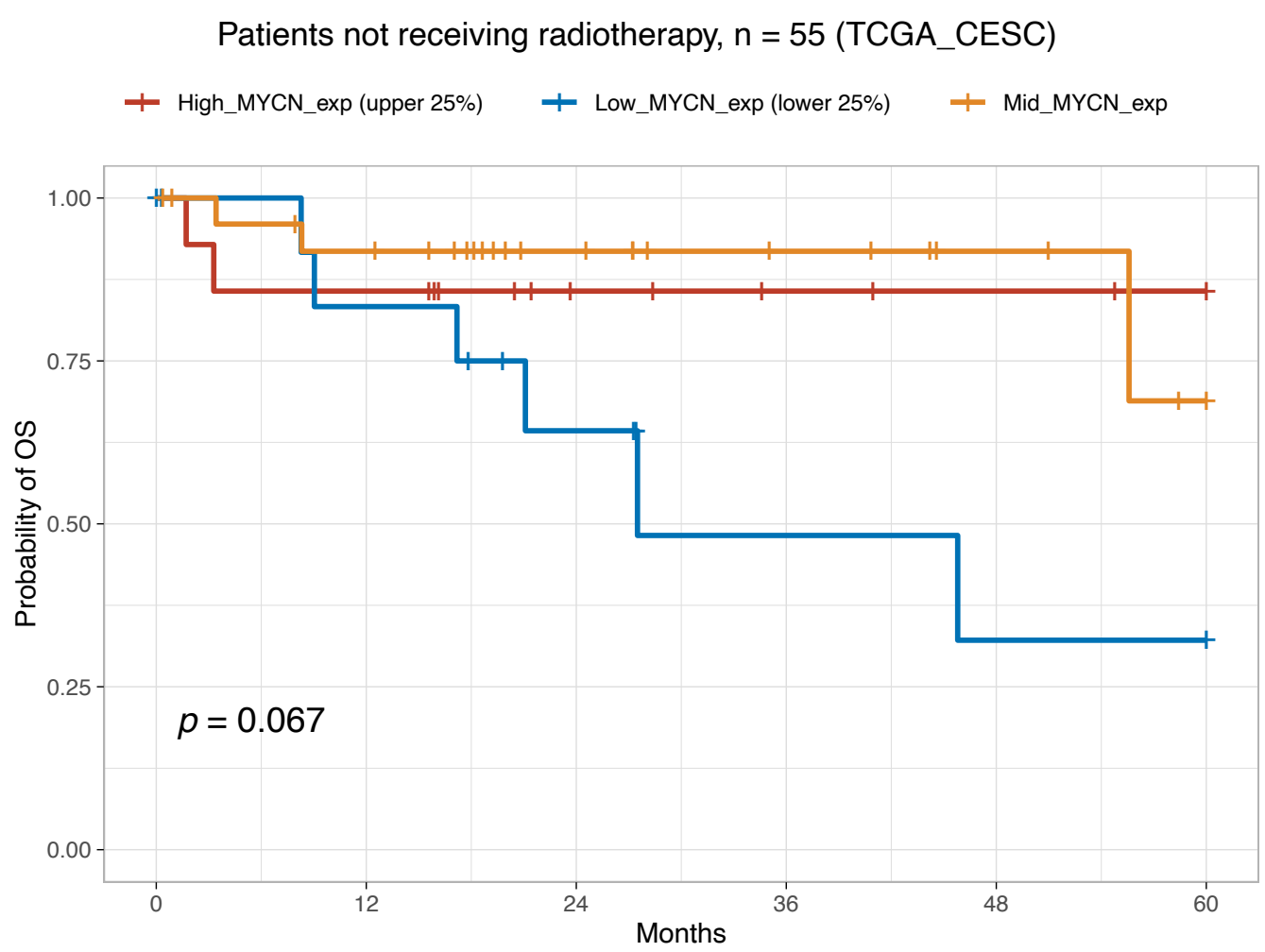

Number at risk

	0	12	24	36	48	60
High_MYCN_exp(upper 25%)	14	12	6	4	3	2
Low_MYCN_exp(lower 25%)	14	10	6	3	2	2
Mid_MYCN_exp	27	22	13	8	5	2

Months

MYCL

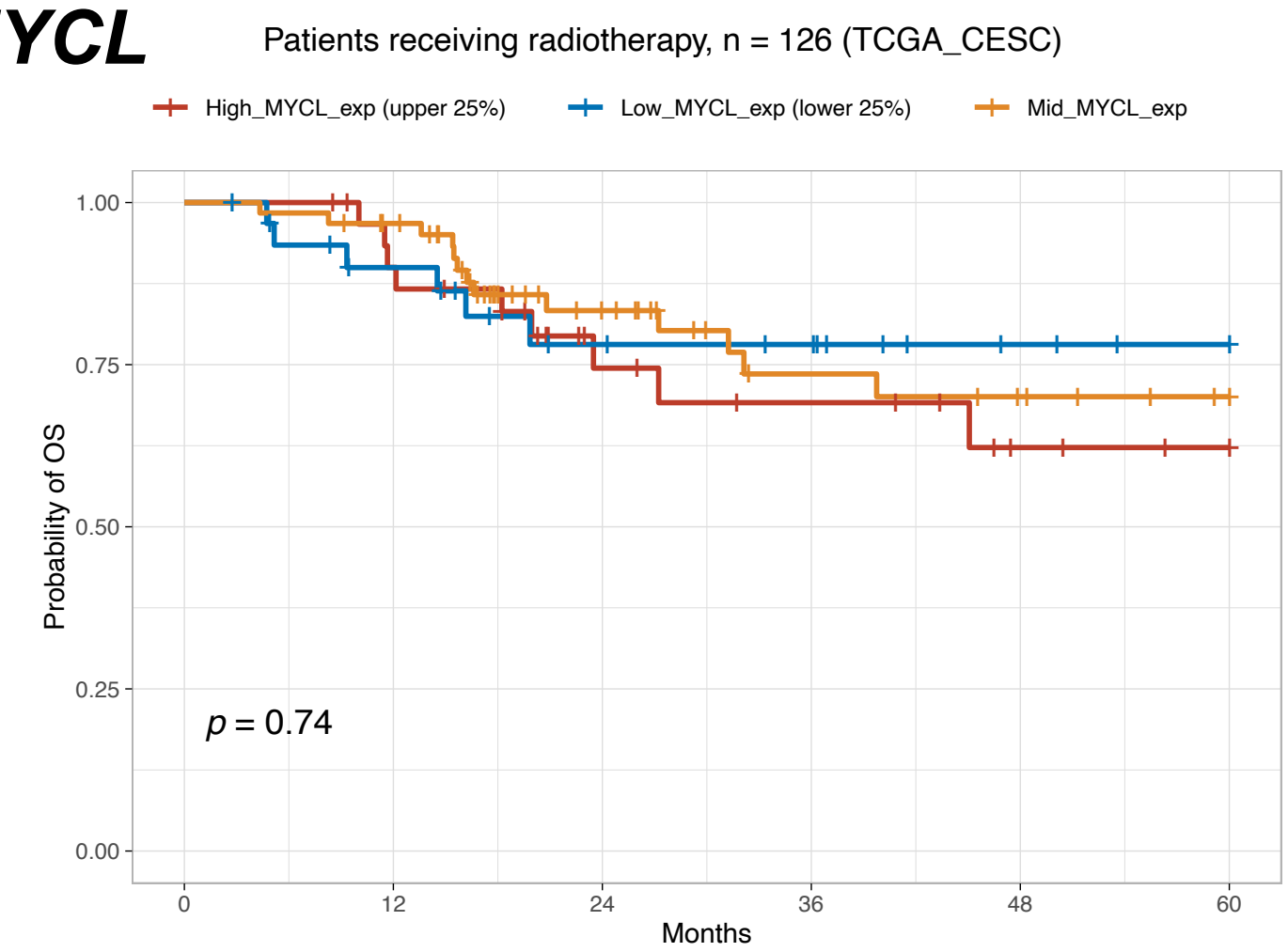

Number at risk

	0	12	24	36	48	60
High_MYCL_exp(upper 25%)	32	27	15	12	7	5
Low_MYCL_exp(lower 25%)	32	25	17	15	9	7
Mid_MYCL_exp	62	57	32	21	18	14

Months

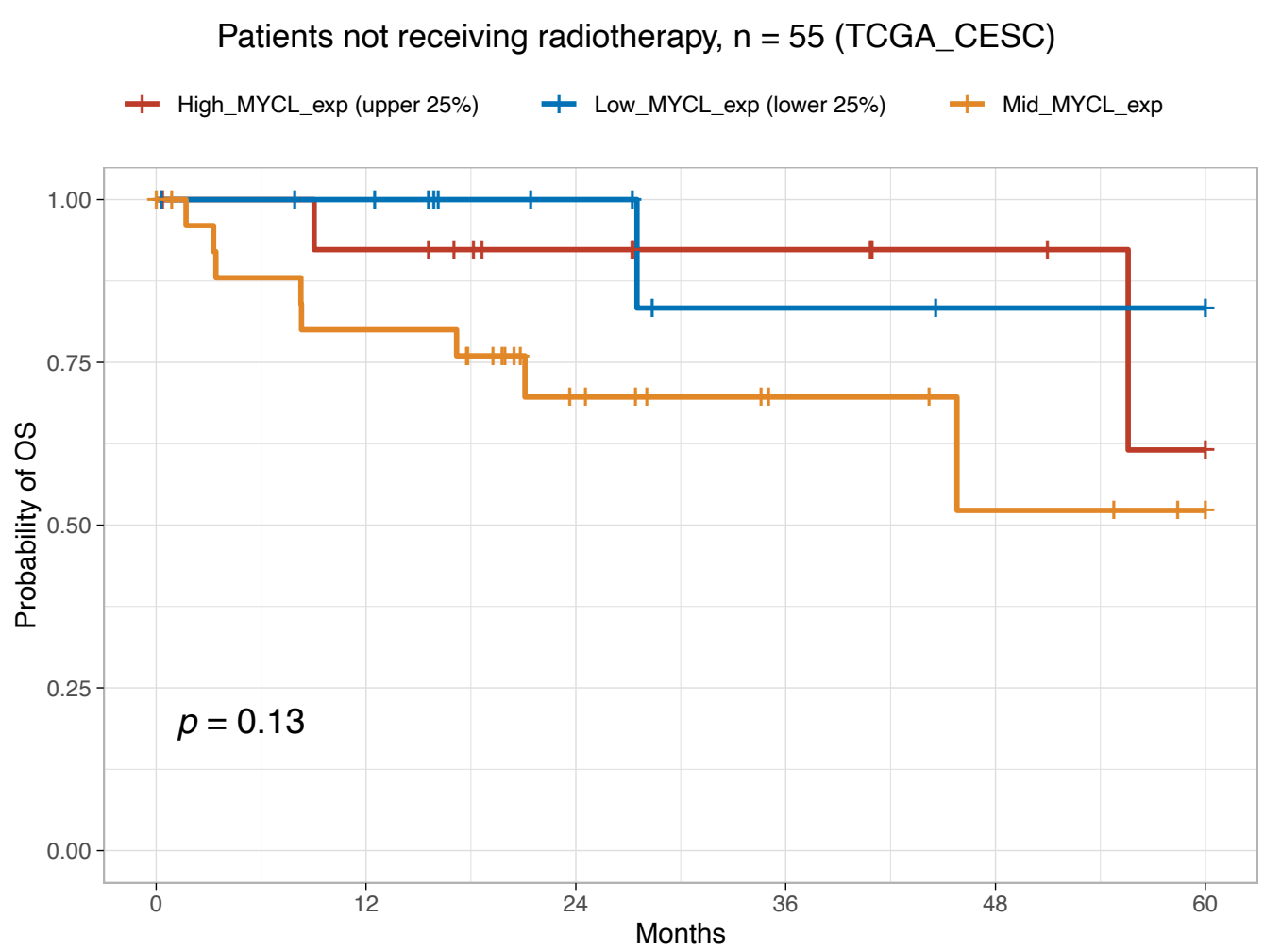

Number at risk

	0	12	24	36	48	60
High_MYCL_exp(upper 25%)	14	12	8	6	4	2
Low_MYCL_exp(lower 25%)	14	12	7	4	3	3
Mid_MYCL_exp	27	20	10	5	3	1

Months
